# Magnetic resonance imaging and BMB score in the evaluation of bone involvement in Gaucher’s disease patients*

**DOI:** 10.1590/0100-3984.2014.0068

**Published:** 2015

**Authors:** Ricardo Andrade Fernandes de Mello, Melissa Bozzi Nonato Mello, Laís Bastos Pessanha

**Affiliations:** 1 PhD, Associate Professor of Radiology, Universidade Federal do Espírito Santo (UFES), Vitória, ES, Brazil.; 2 Master, MD, Hematologist, Centro Capixaba de Oncologia (Cecon), Vitória, ES, Brazil.; 3 MD, Resident in Radiology, Universidade Federal do Espírito Santo (UFES), Vitória, ES, Brazil.

**Keywords:** Gaucher’s disease, Magnetic resonance imaging, Musculoskeletal system, Hematologic diseases

## Abstract

**Objective:**

To evaluate by magnetic resonance imaging changes in bone marrow of patients undergoing treatment for type I Gaucher’s disease.

**Materials and Methods:**

Descriptive, cross-sectional study of Gaucher’s disease patients submitted to 3 T magnetic resonance imaging of femurs and lumbar spine. The images were blindly reviewed and the findings were classified according to the semiquantitative bone marrow burden (BMB) scoring system.

**Results:**

All of the seven evaluated patients (three men and four women) presented signs of bone marrow infiltration. Osteonecrosis of the femoral head was found in three patients, Erlenmeyer flask deformity in five, and no patient had vertebral body collapse. The mean BMB score was 11, ranging from 9 to 14.

**Conclusion:**

Magnetic resonance imaging is currently the method of choice for assessing bone involvement in Gaucher’s disease in adults due to its high sensitivity to detect both focal and diffuse bone marrow changes, and the BMB score is a simplified method for semiquantitative analysis, without depending on advanced sequences or sophisticated hardware, allowing for the classification of the disease extent and assisting in the treatment monitoring.

## Abstract (pt)

**Objetivo:**

Avaliar, por meio de ressonância magnética, alterações da medular óssea de pacientes em tratamento para doença de Gaucher tipo I.

**Materiais e Métodos:**

Estudo descritivo e transversal de pacientes com doença de Gaucher tipo I, com realização de ressonância magnética de 3 T da coluna lombar e fêmures, analisada por meio do escore semiquantitativo denominado *bone marrow burden* (BMB), sem conhecimento das informações clínicas.

**Resultados:**

Dos sete pacientes avaliados (três homens e quatro mulheres), todos apresentaram sinais de infiltração da medula óssea. Osteonecrose da cabeça femoral foi encontrada em três pacientes, deformidade em frasco de Erlenmeyer em cinco e nenhum paciente apresentou colapso de corpo vertebral. A pontuação média do escore BMB total foi 11, variando de 9 a 14.

**Conclusão:**

A ressonância magnética é, atualmente, o método de escolha em adultos para avaliar o envolvimento ósseo na doença de Gaucher, em virtude da sua elevada sensibilidade na detecção de alterações tanto focais quanto difusas da medula óssea, sendo o escore BMB um método simplificado de análise semiquantitativa, sem depender de sequências avançadas ou de *hardware* sofisticado, permitindo classificar a extensão do acometimento da doença e ajudando no monitoramento do tratamento.

## INTRODUCTION

Gaucher’s disease is a hereditary deficiency of the lysosomal glucocerebrosidase enzyme (or beta-glucosidase) which hydrolizes the glucocerebroside glucosylceramide into glucose and ceramide. Such a deficiency leads to deposition of that glycolipid and unleashes histological changes noticeable particularly in the organs that are rich in elements of the monocytic-phagocytic immune system (liver, spleen, lymph nodes and bone marrow)^(1)^.

Gaucher’s disease is classified into three types (I, II, and III) based on the presence and severity of neurological involvement^(1,2)^. Type I (non-neuropathic form) is the most frequently found, corresponding to 95% of all cases, with an incidence of 1:10,000 to 1:20,000^(2,3)^.

Skeletal involvement is seen at radiography in almost all patients, many of which are frequently asymptomatic. Bone involvement severity and the progression rate vary considerably in Gaucher’s disease, but it is generally more aggressive in those patients who present with symptoms in their childhood. Osteopenia, osteonecrosis, osteosclerosis, pathological fracture and vertebral collapse can be associated with Gaucher’s disease^(4)^.

Although the progression of many of these complications may be interrupted or reversed by enzyme replacement therapy, osteonecrosis, osteosclerosis and vertebral compression may be irreversible. Therefore, an early and routine monitoring of the skeletal involvement is imperative^(5-7)^.

Different techniques have been utilized with that purpose, including radiography, computed tomography (CT), magnetic resonance imaging (MRI) and bone scintigraphy. New qualitative techniques and quantitative applications are being tried out in Gaucher’s disease, such as double-beam x-ray absorptiometry, quantitative CT and quantitative MRI techniques, such as spectroscopy^(6,8)^.

## MATERIALS AND METHODS

Images from both femora and lumbar spine were obtained from seven patients presenting with type I Gaucher’s disease in a 3T Achieva MRI scanner (Philips Healthcare; Best, The Netherlands), without intravenous administration of paramagnetic contrast. The TSE T1- and T2-weighted sequences from the lumbar spine were acquired in the sagittal plane, and the TSE T1- and T2-weighted sequences with fat saturation were acquired from both femora in the coronal plane.

The semiquantitative bone marrow burden (BMB) scoring system was utilized to evaluate the MRI studies^(5)^. Such a scoring system is a combination of scores from the peripheral skeleton (femora) and axial skeleton (lumbar spine), based on two characteristics, as follows: signal intensity of altered areas and sites of disease involvement. In the femora, three involvement sites are considered: proximal epiphysis, metaphysis and distal epiphysis (Table 1). In the spine, two patterns are analyzed: patchy infiltration and diffuse infiltration, either with or without involvement of the fat surrounding the basivertebral vein (Table 2). The scores from the lumbar spine and from both femora are summed up, resulting in a maximum total of 16 points (eight from the femora and eight from the lumbar spine).

**Table 1:** BMB scoring for the femora

A: Signal intensity at MRI		
MR sequence	Signal intensity*	Scoring
T2	Hyperintense	2
T2	Mildly hyperintense	1
T2	Isointense	0
T2	Mildly hypointense	1
T2	Hypointense	2
T2	Mixed type	3
T1	Mildly hyperintense or isointense	0
T1	Mildly hypointense	1
T1	Hypointense	2
B: Sites of involvement		
Extent	Scoring	
Diaphysis	1	
Proximal epiphysis/apophysis	2	
Distal epiphysis	3	

* In relation to signal intensity from subcutaneous tissue.

**Table 2:** BMB score in the lumbar spine.

A: Signal intensity at MRI		
MR sequence	Signal intensity*	Scoring
T2	Hyperintense	2
T2	Mildly hyperintense	1
T2	Isointense	0
T2	Mildly hypointense	1
T2	Hypointense	2
T1	Mildly hyperintense	0
T1	Isointense	1
T1	Mildly hypointense	2
T1	Hypointense	3
B: Sites of involvement		
Patchy		Scoring
Sparse		1
Diffuse		2
Absence of fat in the region of the basivertebral vein		3

* In relation to signal intensity from a healthy intervertebral disc.

*Obs.:* A higher score means a more severe bone marrow involvement.

The images of each patient were evaluated by a radiologist experienced in osteoarticular radiology, who was blinded to the clinical data, utilizing the modified BMB scoring system, with the purpose of determining the bone involvement extent.

## RESULTS

**Figure 1:**
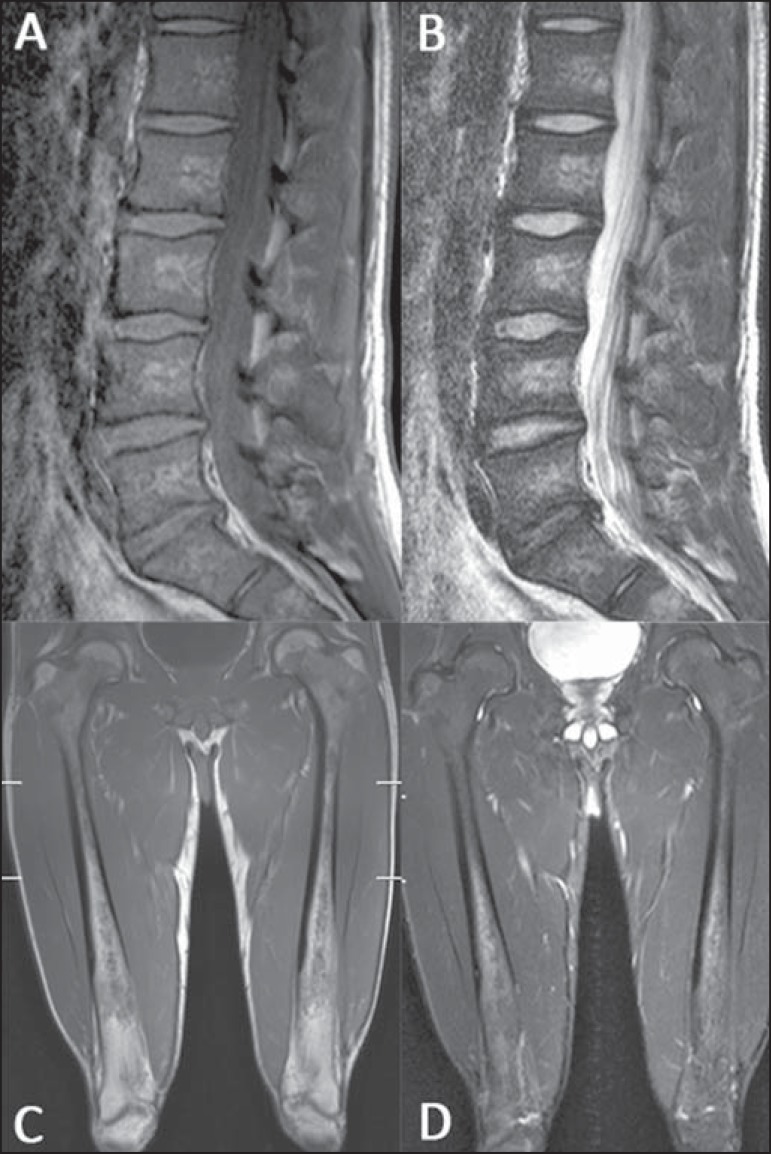
MRI of a male, 28-year-old patient presenting with Gaucher’s disease. T1-weighted (**A**) and T2-weighted (**B**) images of the lumbar spine demonstrate lower signal of the bone marrow in relation to intervertebral discs and presacral fat. The signal intensity score was 4 and for distribution the score was 2, as the fat surrounding basivertebral veins was not replaced, leading to a BMB score of 6 for the lumbar spine. Coronal T1-weighted (**C**) and T2-weighted (**D**) images of the femora were classified as score 3 for signal intensity and 1 for involvement extent, as epiphyseal infiltration was not detected, resulting in a femoral BMB score of 4.

**Figure 2:**
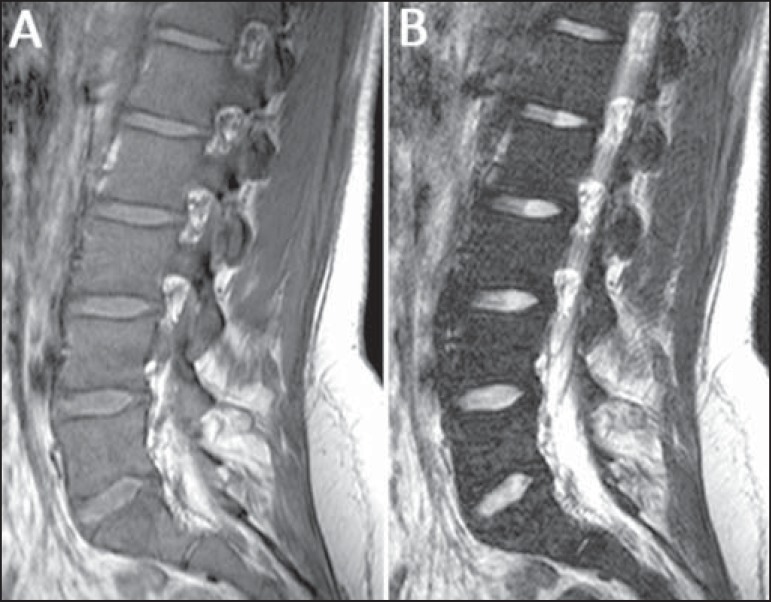
MRI of a female, 22-year-old patient recently diagnosed with Gaucher’s disease. Sagittal T1-weighted (**A**) and T2-weighted (**B**) images of the lumbar spine demonstrate lower signal from the bone marrow in relation to intervertebral discs and presacral fat, including, in this case, replacement of fat surrounding the basivertebral veins (compare with Figure 1, images **A** and **B**) resulting in a score of 4 for signal intensity and 3 for distribution, with a total score of 7 for the lumbar spine.

**Figure 3:**
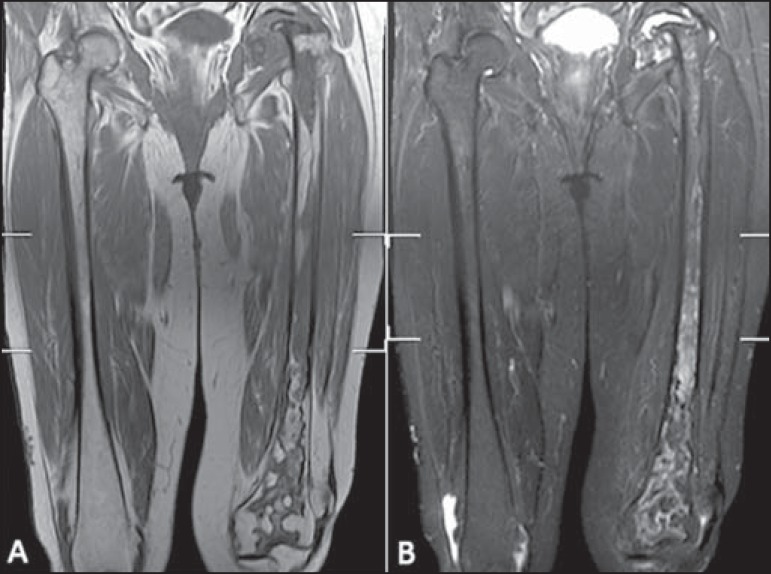
MRI images of a 63-year-old woman with Gaucher’s disease. Coronal T1-weighted (**A**) and T2-weighted (**B**) sequences from both femora were classified as score 5 for signal intensity and 3 for involvement extent, considering the presence of proximal and distal epiphyseal infiltration resulting in a total femoral BMB score of 8.

Bone marrow infiltration was found in all seven patients, three men and four women, with ages ranging between 18 and 69 years (Figures 1, 2 and 3).

**Figure 4:**
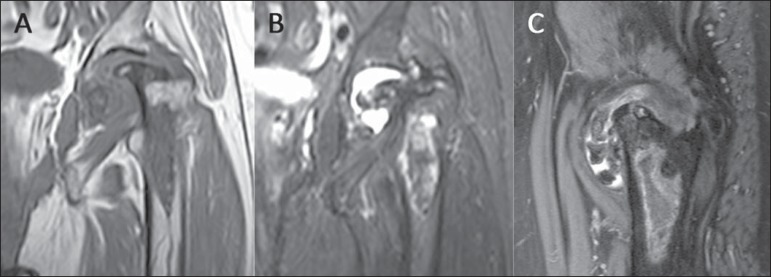
MRI of the same patient on Figure 3. Coronal T1-weighted (**A**), T2-weighted SPAIR (**B**) and sagittal T2-weighted SPAIR (**C**) images of the left hip showing extensive avascular necrosis of the femoral head, with advanced secondary degenerative arthritis.

Femoral head osteonecrosis was found in three patients (Figure 4) and bone marrow infarction was found in four patients. Erlenmayer flask deformity was observed in five cases, and none of the patients presented with vertebral collapse or pathologic femoral fracture. The mean total BMB score (femora and lumbar spine) was 11, ranging from 9 to 14.

## DISCUSSION

The MRI evaluation of the musculoskeletal system has been the subject of a series of recent publications in the Brazilian radiological literature^(9-17)^. In Gaucher’s disease, the bone involvement may not reflect the disease in other organs, with progressive and severe skeletal disease occurring in patients with minor or even absent visceral and hematologic involvement^(18-20)^.

The evaluation and monitoring of bone compromising constitute an important element in the management of Gaucher’s disease, with the objective of instituting a treatment before the onset of irreversible complications such as infarction and avascular necrosis^(11)^. It has been shown that the risk of infarction and avascular necrosis is reduced in patients who start enzyme replacement therapy within two years after diagnosis, as compared with those who experience delays of more than two years between diagnosis and treatment.^(19,20)^

MRI is the method of choice to evaluate the skeleton in Gaucher’s disease in adults, due to its high sensitivity for detecting both focal and diffuse lesions, such as acute bone infarction, trauma, avascular necrosis, infection and infiltration of bone marrow by Gaucher’s cells^(5,6)^.

As Gaucher’s cells infiltrate into the bone marrow, the affected areas will present with hypointense signal at MRI T1- and T2-weighted sequences, due to the replacement of the bone marrow fatty content^(3,5,6,8)^. The pattern of bone infiltration by Gaucher’s cells also provides data on the disease severity; and areas where the signal intensity is not homogeneously decreased are associated with a higher degree of disease irreversibility as compared with areas where the signal intensity is homogeneously decreased^(9,10)^. In studies using MRI, several semiquantitative and quantitative evaluation methods of Gaucher’s disease have been described; and currently the most utilized method is the BMB scoring system, for its simple application in the routine clinical practice^(5,6,8)^.

## CONCLUSION

The BMB scoring system is a widely available, simplified method for semiquantitative evaluation of bone involvement in patients presenting with Gaucher’s disease, relying only on images from the lumbar spine and femora and based on conventional MRI sequences, not depending upon sophisticated hardware and special softwares.

## References

[1] Rosenbloom BE, Weinreb NJ (2013). Gaucher disease: a comprehensive review. Crit Rev Oncog.

[2] Hermann G, Pastores GM, Abdelwahab IF (1997). Gaucher disease: assessment of skeletal involvement and therapeutic
responses to enzyme replacement. Skeletal Radiol.

[3] Wenstrup RJ, Roca-Espiau M, Weinreb NJ (2002). Skeletal aspects of Gaucher disease: a review. Br J Radiol.

[4] Stowens DW, Teitelbaum SL, Kahn AJ (1985). Skeletal complications of Gaucher disease. Medicine (Baltimore).

[5] Maas M, van Kuijk C, Stoker J (2003). Quantification of bone involvement in Gaucher disease: MR imaging bone
marrow burden score as an alternative to Dixon quantitative chemical shift MR
imaging - initial experience. Radiology.

[6] Robertson PL, Maas M, Goldblatt J (2007). Semiquantitative assessment of skeletal response to enzyme replacement
therapy for Gaucher's disease using the bone marrow burden score. AJR Am J Roentgenol.

[7] Mendonça VF, Paula MTM, Fernandes C (2001). Skeletal manifestations in Gaucher's disease. Radiol Bras.

[8] Maas M, Hollak CE, Akkerman EM (2002). Quantification of skeletal involvement in adults with type I Gaucher's
disease: fat fraction measured by Dixon quantitative chemical shift imaging as a
valid paremeter. AJR Am J Roentgenol.

[9] Simão MN, Helms CA, Richardson WJ (2012). Magnetic resonance imaging of disc-related epidural cysts in
nonsurgical and postoperative microdiscectomy patients. Radiol Bras.

[10] Tavares WC, Faria FM, Figueiredo R (2012). Bone attrition: a case of knee pain in osteoarthritis. Radiol Bras.

[11] Moura MVT (2012). Trapped periosteum in a distal femoral physeal injury: magnetic
resonance imaging evaluation. Radiol Bras.

[12] Gomes LM, Lopes FAR, Renck DV (2012). Primary bone lymphoma simultaneous to osteochondroma simulating
sarcomatous degeneration: case report. Radiol Bras.

[13] Nakamura SA, Lorenzato MM, Engel EE (2013). Incidental enchondromas at knee magnetic resonance imaging:
intraobserver and interobserver agreement and prevalence of imaging
findings. Radiol Bras.

[14] Souza CGD, Gasparetto EL, Marchiori E (2013). Pyogenic and tuberculous discitis: magnetic resonance imaging findings
for differential diagnosis. Radiol Bras.

[15] Machado BB, Lima CMAO, Junqueira FP (2013). Magnetic resonance imaging in intersection syndrome of the forearm:
iconographic essay. Radiol Bras.

[16] Canella C (2013). Dynamic gadolinium injection in the assessment of enchondromas
[Editorial]. Radiol Bras.

[17] Terazaki CRT, Trippia CR, Trippia CH (2014). Synovial chondromatosis of the shoulder: imaging
findings. Radiol Bras.

[18] Poll LW, Koch JA, vom Dahl S (2001). Magnetic resonance imaging of bone marrow changes in Gaucher disease
during enzyme replacement therapy: first German long-term results. Skeletal Radiol.

[19] Cox TM, Aerts JM, Belmatoug N (2008). Management of nonneuronopathic Gaucher disease with special reference
to pregnancy, splenectomy, bisphosphonate therapy, use of biomarkers and bone
disease monitoring. J Inherit Metab Dis.

[20] Terk MR, Dardashti S, Liebman HA (2000). Bone marrow response in treated patients with Gaucher disease:
evaluation by T1-weighted magnetic resonance images and correlation with reduction
in liver and spleen volume. Skeletal Radiol.

